# Spatial attention and representation of time intervals in childhood

**DOI:** 10.1038/s41598-020-71541-6

**Published:** 2020-09-11

**Authors:** Barbara Magnani, Alessandro Musetti, Francesca Frassinetti

**Affiliations:** 1 Centro INforma-MEnte, Via Brigata Reggio 32, 42124 Reggio Emilia, Italy; 2 Department of Humanities, Social Sciences and Cultural Industries, University of Parma, Parma, Italy; 3 Department of Psychology, University of Bologna, Bologna, Italy; 4 Maugeri Clinical Scientific Institutes - IRCCS of Castel Goffredo, Castel Goffredo, Mantova Italy

**Keywords:** Neuroscience, Psychology

## Abstract

Spatial attention and spatial representation of time are strictly linked in the human brain. In young adults, a leftward shift of spatial attention by prismatic adaptation (PA), is associated with an underestimation whereas a rightward shift is associated with an overestimation of time both for visual and auditory stimuli. These results suggest a supra-modal representation of time left-to-right oriented that is modulated by a bilateral attentional shift. However, there is evidence of unilateral, instead of bilateral, effects of PA on time in elderly adults suggesting an influence of age on these effects. Here we studied the effects of spatial attention on time representation focusing on childhood. Fifty-four children aged from 5 to 11 years-old performed a temporal bisection task with visual and auditory stimuli before and after PA inducing a leftward or a rightward attentional shift. Results showed that children underestimated time after a leftward attentional shift either for visual or auditory stimuli, whereas a rightward attentional shift had null effect on time. Our results are discussed as a partial maturation of the link between spatial attention and time representation in childhood, due to immaturity of interhemispheric interactions or of executive functions necessary for the attentional complete influence on time representation.

## Introduction

The purpose of the present work is to study the functional link between spatial attention and the spatial representation of time in children. The study of how the spatial representations are modulated by shifting spatial attention has allowed a finer and finer comprehension of the cognitive features of spatial representations. Regard the representation of magnitudes (as numbers or time) the literature converges that it is cognitively structured as a line ascending ordered from left to right and that a shift of spatial attention modifies such a representation according to the side of the attentional shift^1^.

One of the main techniques to obtain a lateralized shift of spatial attention is prismatic adaptation (PA)^2^. This technique is widely used because it induces an involuntary, endogenous and covert attentional shift toward a side of space by means of a visuo-motor adaptation procedure^3–5^. Even if the PA procedure is principally visuo-motor, the effects of PA are widely demonstrated on high-order spatial representations^6–9^. In healthy adults, the effects of a leftward and a rightward shift by PA on both visual and auditory temporal intervals with a paradigm in which subjects verbally judged stimuli durations were investigated^10^. Authors found that a leftward attentional shift modifies time representations toward an underestimation and a rightward attentional shift toward an overestimation^11,12^ both for visual and auditory stimuli^10^. These results suggested that a shift of attention by PA, in both left and right direction, induces a modification of time spatial representation at a supra-modal level^10^.

An explanation of these effects is that the attentional shift provoked by the visuo-motor adaptation procedure enhances the cortical excitability of the hemisphere contralateral to the side of the shift. Such an enhance of hemispherical excitability, that in adulthood can be obtained specularly for both hemispheres, would induce a cognitive reorganization of the contralateral part of the spatial representation^13,14^.

However, these symmetrical effects of PA deviations were not found in elderly subjects. In participants with an average age of 65 years-old, a leftward attentional shift induced an underestimation of time while a rightward attentional shift was ineffective on time^15^. This asymmetry can be explained by a faster age-related decline of the right than the left hemisphere^16^ that can result in a hemispheric unbalanced excitability affecting spatial representations. In support, spatial bias are frequently demonstrated in elderly^17^ in the visual attention orientation^18^, in line bisection task^19^ and in mental time travel task that involves a spatial representation of time^20^. Since PA acts rebalancing asymmetric deficit of spatial attention (see PA effects on time in neglect patients^13^), in the case of unbalanced hemispheric excitability due to a right hemispheric decline, PA could enhance the excitability of the right less efficient hemisphere (by a leftward attentional shift).

Following data on PA effects on time in young and elderly adults, the aim of the present study is to investigate the effects of PA on time across childhood when attentional processes, spatial cognition and time processing are not yet completely developed in all their components^21,22^. Even if we have evidence that infants adapt to prisms^23^ and that they represent time as a line ascending ordered from left to right^24,25^, the effects of a shift of spatial attention by PA on such representation are unknown in this population. The investigation of the functional link between spatial attentional shift and time representation in children can integrate data on attention and time in children and adults shedding light on maturational implications of such a link and, taken together with elderly persons data, of hemispherical lateralization for spatial representation of time in the lifespan.

To investigate PA effects on time in childhood, participants from 5 to 11 years old, attended a time bisection task with verbal response, on visual and auditory stimuli before and after a session of (PA) with a rightward and leftward deviation inducing, respectively, a leftward or a rightward shift of attention. First of all, accordingly with literature, performance in the temporal bisection task is expected to improve from youngest to oldest children^26,27^. Second, regarding PA effects on time, if the functional link between spatial attention and time representation is mature, an underestimation or overestimation after a leftward or rightward attentional shift respectively, for visual and auditory stimuli, is expected. By contrast, if the link between spatial attention and time representation is not complete, due to immature anatomical and functional mechanisms subtending these functions, differences in ages, spatial attentional shift and/or modalities are expected.

## Results

### Effect of prismatic adaption on time bisection task: point of subjective equality (PSE)

#### Leftward aftereffect

**Figure 1:**
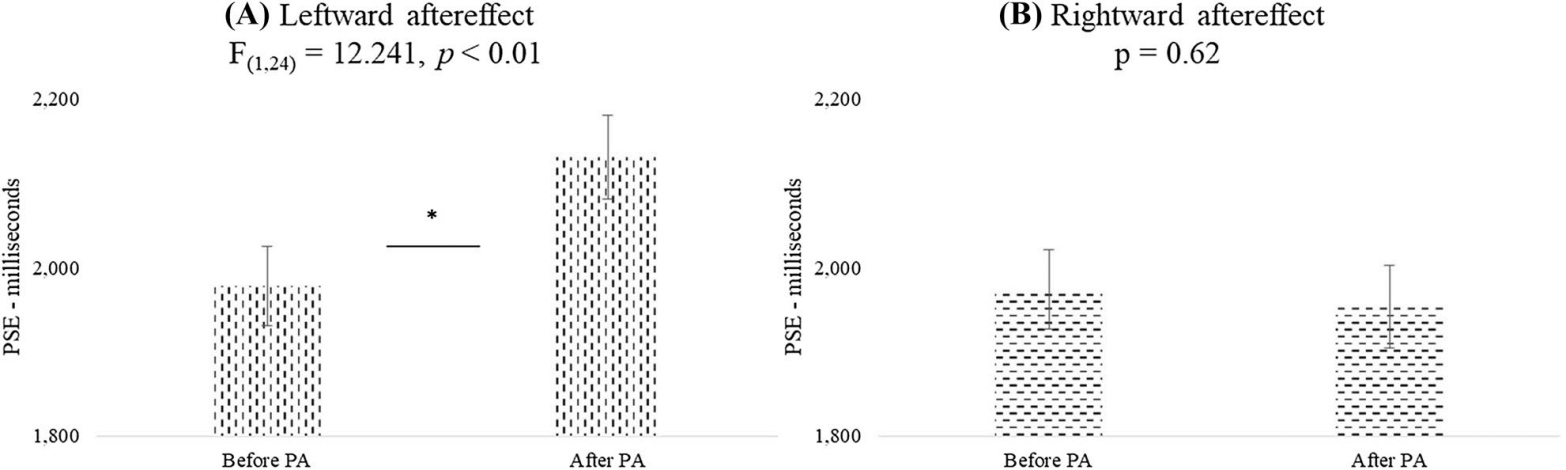
The graph represents the PSE mean values of Condition (Before PA; After PA) for the analysis on the leftward (**A**) and rightward (**B**) aftereffect. Error bars indicate standard errors of means. *Symbol indicates *p *< 0.05.

The ANOVA on PSE with Age as between-groups variable and Modality and Condition as within-subject variables, revealed a significant effect of Condition [F_(1,24)_ = 12.241, *p* < 0.01, *ηp*^2^ = 0.338] indicating an *underestimation of time after-PA relative to before-PA* (2131 ms vs 1978 ms, see Fig. 1A). The effect of Age (*p* = 0.89), its interaction with Condition (*p* = 0.56) or Modality (*p* = 0.49), the interaction Condition x Modality (*p* = 0.84) and the threefold interaction (*p* = 0.59) were not significant indicating a similar influence of the leftward aftereffect on time for all ages and modalities.

#### Rightward aftereffect

The ANOVA revealed no significant effect of Condition (*p* = 0.62 see Fig. 1B for values), Age (*p* = 0.93) or interactions among variables (*p* > 0.21 for all comparisons) indicating that *the rightward aftereffect does not induce any modulation of time* in any group or modality.

When the leftward and rightward aftereffects were compared by an ANOVA with Aftereffect and Age as between-groups variables and Modality and Condition as within-subjects variables the main effect of Modality [F_(1,48)_ = 6.412, *p* < 0.05, *ηp*^2^ = 0.118] was significant. Visual stimuli (2054 ms) were underestimated relative to auditory stimuli (1963 ms). Condition [F_(1,48)_ = 4.792, *p* < 0.05, *ηp*^2^ = 0.091] and the interaction between Condition and Aftereffect [F_(1,48)_ = 8.312, *p* < 0.01, *ηp*^2^ = 0.148] were also significant. Values confirmed an increase of PSE after the leftward (2131 ms) but not rightward (1954 ms) aftereffect relative to before PA (leftward: 1978 ms; rightward: 1974 ms). No other interactions were significant (*p* > 0.13 for all comparisons).

### Effect of prismatic adaption on accuracy in time interval discrimination: Weber ratio (WR)

#### Leftward aftereffect

**Figure 2:**
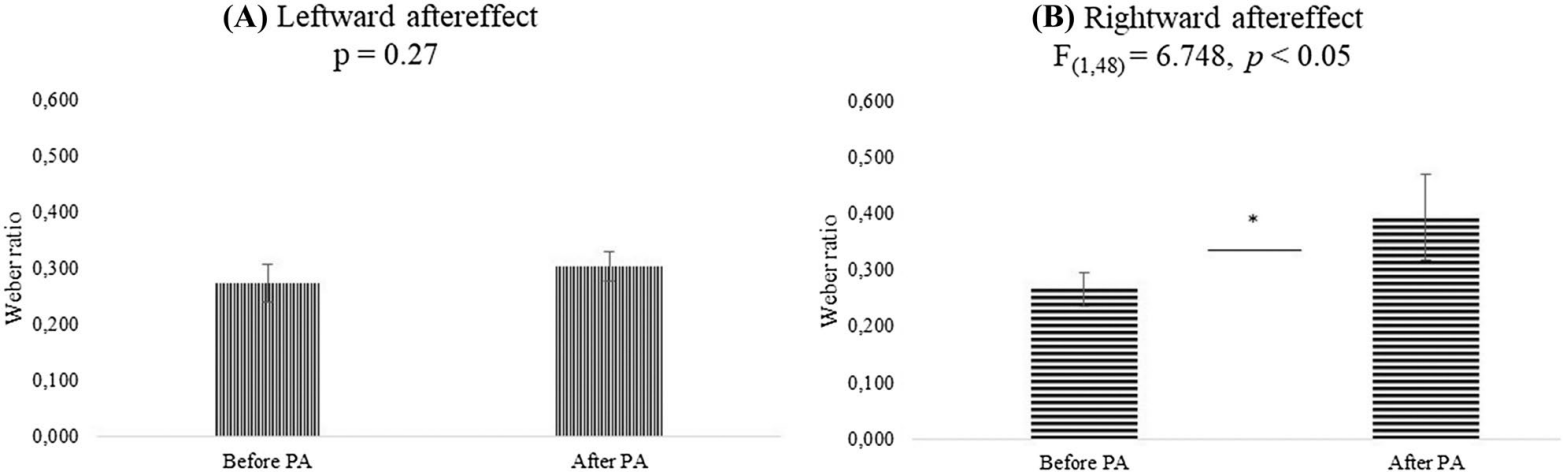
The graph represents the Weber ratio mean values of Condition (Before PA; After PA) for the analysis on the leftward (**A**) and rightward (**B**) aftereffect. Error bars indicate standard errors of means. *Symbol indicates *p* < 0.05.

The variable Age was significant [F_(2,24)_ = 8.134, *p* < 0.01, *ηp*^2^ = 0.404], showing a better time discrimination with increasing age, since WR was lower in late-children (0.207) than in children (0.294) and in early-children (0.366, *p* < 0.001 for all comparisons). The effect of Modality was also significant [F_(1,24)_ = 13.151, *p* < 0.01, *ηp*^*2*^ = 0.354]: time discrimination was better for auditory (0.247) than for visual stimuli (0.332). No significant effects of Condition (*p* = 0.27, Fig. 2A for values) and interactions among variables were found (*p* > 0.29 for all comparisons).

#### Rightward aftereffect

Again, the variable Age was significant [F_(2,24)_ = 12.597, *p* < 0.001, *ηp*^2^ = 0.512], showing a better time discrimination with increasing age, since WR was lower in late-children (0.176) than in children (0.327) and in early-children (0.489, *p* < 0.001 for all comparison). The effect of Modality was also significant [F_(1,24)_ = 8.788, *p* < 0.01, *ηp*^2^ = 0.268]: time discrimination was better for auditory (0.260) than for visual stimuli (0.402). An interesting significant effect of Condition was found [F_(1,24)_ = 6.748, *p* < 0.05, *ηp*^2^ = 0.219] since time interval discrimination was worst after PA than before PA (0.395 vs 0.266 see Fig. 2B).

When the leftward and rightward aftereffects were compared by an ANOVA with Aftereffect and Age as between-groups variables and Modality and Condition as within-subjects variables, no interactions among variables were significant (*p* > 0.08).

### Prismatic adaptation: error reduction and aftereffect

#### Error reduction

**Figure 3:**
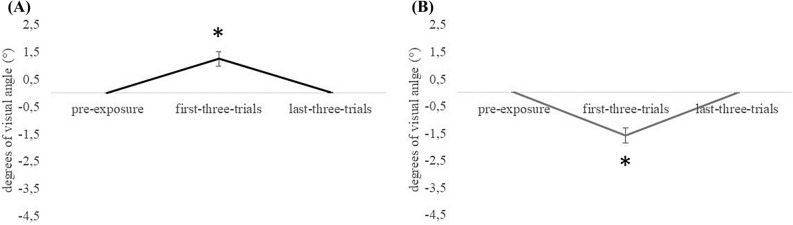
Visible pointing. Error reduction. Displacement of the visible pointing, expressed in degrees of visual angle (°), for right (**A**) and left (**B**) prismatic deviation in the pre-exposure condition, in the first three and the last three trials of the exposure condition. Error bars represent standard errors of means. *Symbol indicates *p* < 0.05.

The ANOVA on the right prismatic deviation, inducing a *leftward aftereffect*, revealed a significant effect of Condition [F_(2,23)_ = 11.900, *p* < 0.001, *ηp*^2^ = 0.509] but not of Age or interaction between variables (*p *> 0.71 for all comparisons). The result indicates that, independent on the participants’ age, the pointing displacement in the first three trials of the exposure condition (1.248°) is different from the pointing displacement in the pre-exposure condition (− 0.004°) and in the last three trials of the exposure condition (0.026° see Fig. 3A). Similarly, the ANOVA on the left prismatic deviation, inducing a *rightward aftereffect*, revealed a significant effect of Condition [F_(2,23)_ = 15.776, *p* < 0.001, *ηp*^2^ = 0.578] but not of Age or interaction between variables (*p* > 0.30 for all comparisons). The result indicates that, independent on the participants’ age, the pointing displacement in the first three trials of the exposure condition (− 1.585°) is different from the pointing displacement in the pre-exposure condition (0.007°) and in the last three trials of the exposure condition (− 0.015°, see Fig. 3B).

#### Aftereffect

**Figure 4:**
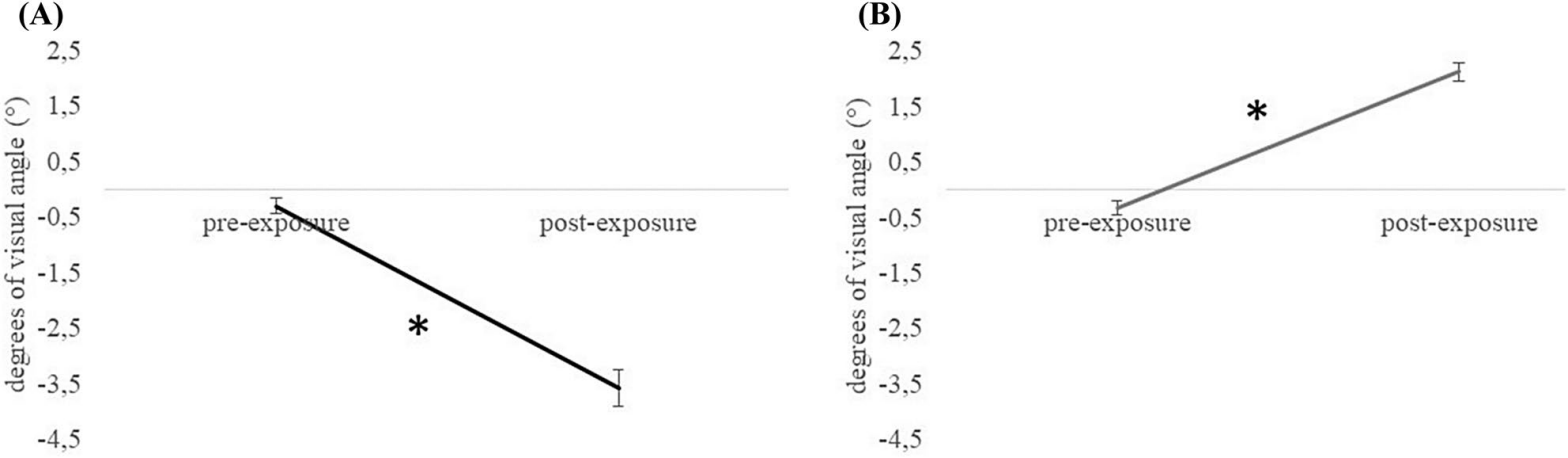
Invisible pointing. Aftereffect. Displacement of the visible pointing, expressed in degrees of visual angle (°), for right and left prismatic deviation inducing a leftward (**A**) and a rightward (**B**) aftereffect respectively, in the pre-exposure and post-exposure condition. Error bars represent standard errors of means. *Symbol indicates *p* < 0.05.

The ANOVA on the *leftward aftereffect* revealed a significant effect of Condition [F_(1,24)_ = 136.500, *p* < 0.001, *ηp*^2^ = 0.850] but not of Age or interaction between variables (*p* > 0.48 for all comparisons). The result indicates that the pointing displacement in the post-exposure condition (− 3.582°) is different from the pointing displacement in the pre-exposure condition (− 0.311° see Fig. 4A).

Similarly, the ANOVA on the *rightward aftereffect* revealed a significant effect of Condition [F_(1,24)_ = 152.810, *p* < 0.001,* ηp*^2^ = 0.864] but not of Age or interaction between variables (*p* > 0.09 for all comparisons). The result indicates that the pointing displacement in the post-exposure condition (2.119°) is different from the pointing displacement in the pre-exposure condition (− 0.330°, see Fig. 4B).

## Discussion

Here we investigated whether the functional link between spatial attention and spatial representation of time is complete in childhood as in adulthood. Specifically, we investigated whether a shift of spatial attention obtained by a visuo-motor adaptation procedure (PA) affects a supra-modal left-to-right representation of time intervals. Children were submitted to a time bisection task with verbal response on visual and auditory stimuli before and after a session of PA shifting spatial attention to the left or to the right.

The first, novel and interesting result is that in children from 5 to 11 years-old only the leftward shift of spatial attention induced an underestimation of time whereas a rightward shift had no effect on time. Similarly, in a previous work on children of the same age, an underestimation of time for stimuli presented on the left, but not an overestimation of stimuli presented on the right was found^24^.

Such an asymmetrical lateralized effect is different from the bilateral effects of PA on time found in young adults^10,11^. Interestingly, this result parallels that found in healthy elderly persons, showing an underestimation of time after a leftward attentional shift and the null effect of a rightward attentional shift^15^. Taken together, data on population of different ages suggest that the effects of spatial attention on time representation follows a U slope, from childhood to seniority, with the minimum gradient of asymmetrical lateralized PA effects on time in adulthood. Two not in contrast explanations for this lateralization gradient of PA effects on time in the lifespan can be advanced.

It is possible that PA rebalances the asymmetry of interhemispheric excitability, enhancing the excitability of the right hemisphere^13,14^. This asymmetry could be due to low maturation in childhood, or high decline in seniority, of the right relative to the left hemisphere^28–31^. In this respect, it has been shown that the left hemisphere develops faster than the right one^28^. Interestingly, in the age range from 5 to 11 years old, it is demonstrated an increase of thickness, an indicator of maturation, in the frontal and temporo-parietal areas in the left hemisphere and a spread thinning, an indicator of low maturation, of the frontal and parieto-occipital areas in the right hemisphere. This indicates a preference for the expansion of language networks in this age period instead of spatial attentional networks^28^. Moreover, interhemispheric excitation-inhibition fully functioning processes have been demonstrated in adults but not in children^32^ and aged persons^33^. A speculation could be that in *young-adults* the interhemispheric excitability is symmetric and an attentional shift affects spatial representations activating both the hemispheres. In children and elderly people, the interhemispheric excitability is asymmetric and an attentional shift modulates spatial representations when it activates mainly the right hemisphere. A result of the present work in favor of an effect of PA in the rebalancing of a pre-existent inter-hemispheric unbalance is that the rightward aftereffect induced a worsening of the accuracy in time discrimination (measured as Weber ratio values). Since leftward prismatic lenses inducing rightward aftereffect enhance the left hemisphere excitability^14^, this could result in an enhanced inter-hemispheric unbalance in children. We can speculate that this prismatic deviation, not only has a null effect on the spatial representation of time but also plays against basic time processing making the raw representation of durations noisier^34^ than before PA.

The second explanation can integrate with the latter and refers to the developmental trajectory of executive functions necessary for the manipulation of spatial representations by means of the attentional shift. Children between 5 and 9 years-old and elderly from 59 years-old, present with a low efficiency of executive functions due to a not complete maturation or to a decline of these functions, respectively^17^. The low efficiency in executive functions can fail to contrast the effects on cognitive processes induced by the asymmetric hemispheric excitability. By contrast, in condition of maximum executive efficiency, as in young adults (from 19 to 32 years-old^17^), these effects are reduced. In support, it was found that children, as elderly people, tend to put their attention preferably to the left of space^35,36^ and to bisect lines toward the left relative to the midpoint^37^. More interestingly, children and elderly people showed greater lateralized spatial bias on a number bisection task when the task required the inhibition of a distractor stimulus^38^.

Another worthy finding of the present study is a general tendency to underestimate visual time relative to auditory time. The underestimation of time for visual relative to auditory stimuli in adults as in children is well documented in time literature^26,39–43^. The most accredited interpretation is that the speed of the pacemaker-accumulator module of time processing^44^ is faster for audition than for vision^40^. This means that this module accumulates more pulses in the time unite for auditory than for visual stimuli resulting in a longer estimation judgment of the to-be-timed interval. The accumulation of more pulses in the time unit makes the sensitivity for auditory time higher than for visual time at all ages^43^ as confirmed by our data. What is interesting here is that, despite differences in the rate of pulses accumulation and in the sensitivity for visual and auditory stimuli, a shift of spatial attention modulates time perception regardless of modality in children from 5 to 11 years-old. This favours the hypothesis that PA affects high and not low orders of time representation, that are supra-modal in nature^12^. Our new finding on children extends that found in adults^10^ pointing out the presence of a high-order cognitive spatial representation of time even at an early stage of cognitive development.

Finally, a result that is worthy to discuss is that children’s time interval discrimination accuracy improves with age as widely accepted in literature independent on modality^43^, on stimuli duration range or paradigms adopted^24,34,45^. However, the effects of PA is not different in the three age groups, suggesting that the specific pattern of lateralized effects of PA on time remains stable across all childhood. Although data on adolescents are missing in literature and we just know data on young adults (mean, 25 years-old)^10,11^ it is possible to speculate that, to obtain complete bilateral effects of spatial attention on time representation, the brain needs the complete maturation of executive strategies to contrast asymmetrical hemispheric excitability that would be reached with adolescence neuro-maturation.

## Methods

### Participants

The sample consisted of 54 right-handed children with no diagnosis of neurological or psychological diseases recruited from four infancy and primary schools situated in the northern Italy (Emilia Romagna). Sample size for the analysis (repeated measure ANOVA, within factors) was determined a priori by conducting a power analysis using G*Power 3.0.10. A small to medium effect size (*ηp*^2^ = 0.194) was specified. Within our chosen sample size and effect size, the power (1 − β) was approximately 0.80 and the critical F was 4.030 (see also^46^). The sample was composed by three groups of children with different ages: eighteen 5-to-6 years-old children (early-children-group, 10 boys, age mean = 5.39 years, SD = 0.50 years); eighteen 7-to-8 years-old children (children-group, 6 boys, age mean = 7.22 years, SD = 0.55 years, years of education mean = 1.94, SD = 0.23 years); eighteen 9-to-11 years-old children (late-children, 6 boys, age mean = 10.06 years, SD = 0.87 years, years of education mean = 4.61, SD = 0.61 years). All parents of the involved children signed informed consent and all children were naïve as to the purpose of the study. The study, inserted in a project titled “Time in the developmental age” was approved by the Ethics Committee of the Department of Psychology of Bologna and it is conform to the Declaration of Helsinki.

All participants of the three age groups (early-children, children and late-children) performed the time bisection task for both visual and auditory modality before and after a session of PA. The order of visual and auditory task was counterbalanced among subjects. Half participants were submitted to PA inducing a leftward aftereffect and the other half were submitted to PA inducing a rightward aftereffect. Children were randomly assigned to the “leftward aftereffect” group or to the “rightward aftereffect” group. Comparing the mean age of the leftward and the rightward aftereffect groups, by using 2-tailed independent samples t-test, no significant differences between the two groups emerged (early-children, M_age_ = 5.4 vs 5.3 years, *p* = 0.68; children, M_age_ = 7.2 vs 7.2 years, *p* = 1.00; late-children, M_age_ = 10.0 vs 10.1 years, *p* = 0.78).

### Procedure

#### Time bisection task

Subjects sat facing a Personal Computer, at a distance of 60 cm. Two modalities specific kinds of stimuli were presented. Visual stimuli were black circumferences (diameter = 4 cm) on a white background presented at the centre of the computer screen which got fully black for 5 variable time intervals (1400 ms, 1700 ms, 2000 ms, 2300 ms, 2600 ms). Auditory stimuli were FA tones (349 Hz) during one of 5 variable time intervals (1400 ms, 1700 ms, 2000 ms, 2300 ms, 2600 ms).

In the *training phase,* subjects were initially presented with the “short” interval (1400 ms) and the “long” interval (2600 ms) for one of two modalities (visual or auditory). After the first presentation, they were trained to classify stimuli as “short” or “long” with verbal responses. The training phase concluded when the percentage of correct responses on 20 trials (10 for “short” and 10 for “long” interval) was at least 80%.

In the *test phase,* subjects were presented with all 5 probe-intervals (1400 ms, 1700 ms, 2000 ms, 2300 ms, 2600 ms). In this phase, 50 trials (10 for each time interval) were presented in a random order. Subjects were required to classify stimuli as “short” or “long” with verbal responses.

#### Prismatic adaptation

Subjects were seated at a table in front of a box (height = 30 cm, depth = 34 cm at the centre and 18 cm at the periphery, width = 72 cm) that was open on the side facing the subjects and on the opposite side, facing the experimenter. The experimenter placed a visual target (a pen) at the distal edge of the top surface of the box, in one of three possible positions (randomly determined on each trial): a central position (0°), 21° to the left of centre, and 21° to the right of centre. For each target placement, subjects performed the pointing task. It consisted in keeping their right hand at the level of the sternum and then pointing toward the pen using the index finger of the same hand; the experimenter recorded the end position of the subject’s pointing direction. In the invisible pointing trials, the arm was totally covered by a black sheet and the subjects did not see any part of the trajectory of the arm. In the visible pointing trials, the arm was covered only in the proximal part and the subjects could see the last third of the trajectory of the pointing movement.

The three conditions of the pointing task (as analysed in the Results section) were structured as follow. *Pre-exposure condition*: 60 trials, 30 in visible pointing and 30 in invisible pointing. *Exposure condition:* 90 trials in visible pointing; subjects wore prismatic lenses that induced a 10° shift of the visual field to the right or to the left. *Post-exposure condition*: 30 trials in invisible pointing; immediately after removal of the prisms.

### Statistical analysis

#### Time bisection task

To verify the effects of PA on time bisection, for each prismatic deviation (inducing a leftward or a rightward aftereffect) an ANOVA, on PSE and WR values, was conducted with Age (early-children; children; late-children) as between-groups variable and Modality (visual, auditory) and Condition (before-PA, after-PA) as within-subjects variables. Then, to explore a possible interaction between the condition and aftereffect deviation we also conducted an ANOVA on PSE and WR values with Aftereffect (leftward vs rightward) and Age (early-children; children; late-children) as between-groups variables and Modality (visual, auditory) and Condition (before-PA, after-PA) as within-subjects variables. For all ANOVAs, post-hoc analysis was conducted where necessary with the Duncan test and the effect size was reported as partial eta square.

**Figure 5:**
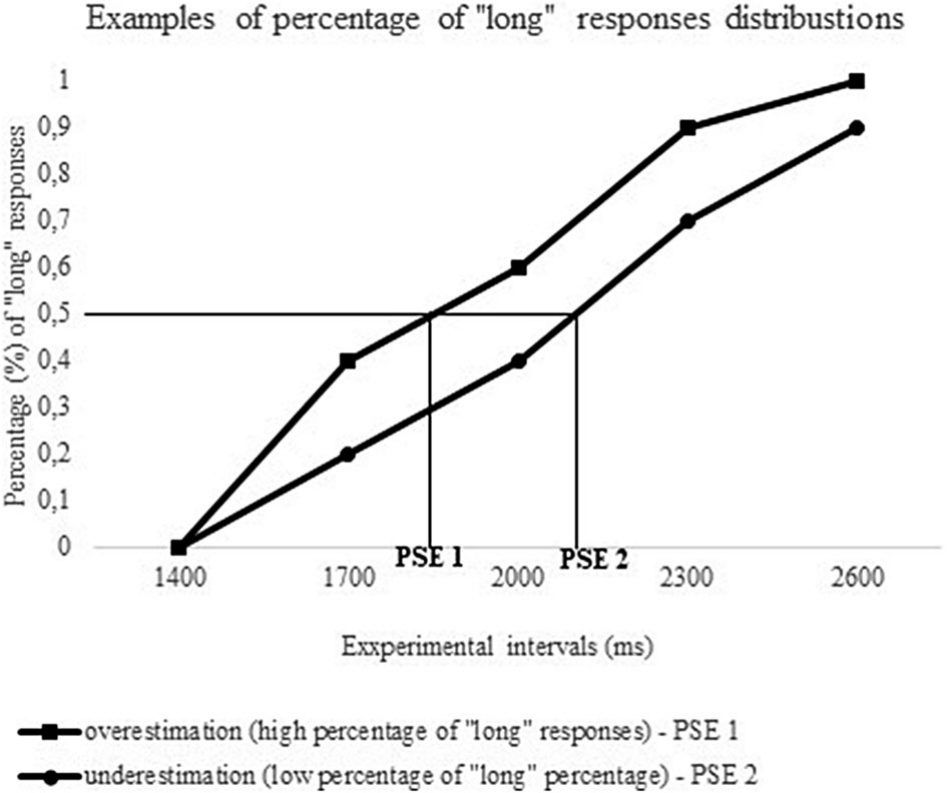
Graphical representation of distributions of percentage (%) of long responses in the five experimental intervals (1400, 1700, 2000, 2300, 2600 ms) expressed in milliseconds (ms). The graph shows that for a distribution of high percentage of “long” responses (overestimation) the point of subjective equality (PSE), indicated as PSE 1, is lower than for a distribution of low percentage of “long” responses (underestimation—PSE 2).

*To calculate PSE*, the percentage of “long” responses for all probe-durations (1400 ms, 1700 ms, 2000 ms, 2300 ms, 2600 ms) was considered for each subject in the visual and auditory temporal bisection task. Then, we plotted the percentage of “long” responses as a function of stimulus durations and, by means of a syntax built on SPSS 25 version, we computed the logistic regression^47^ using the following formula: y = a/(1 + exp(− k*(x − xc)). This allows us to calculate the PSE as the “difference limen”, that is half the difference of the duration classified as "long" on 75% of trials and that classified as "long" on 25% of trials). In more simple words, PSE is a measure of the signal duration at which “short” or “long” responses occur with equal frequency (percentage of “long” responses = 50%). In our paradigm, the objective stimulus duration representing the medium point between the short and long reference duration was 2000 ms. A PSE value below 2000 ms reflects duration overestimation (i.e., durations are perceived longer than they actually are) since the PSE decreases when the percentage of long responses increases. Whereas, a PSE value above 2000 ms reflects duration underestimation (i.e., durations are perceived shorter than they actually are) since the PSE increases when the percentage of long responses decreases (see Fig. 5 for a graphical representation).

*To calculate the WR* values, for each test phase of each subject in the visual and auditory temporal bisection task we plotted the same percentage of “long” responses as a function of stimulus durations as for the PSE calculation. Then, we computed the “different limen” divided by the correspondent PSE. The WR represents a measure of the subjects' accuracy in the discrimination between two stimuli. Here, it is an index of individual’s sensitivity to time that is the ability to discriminate time intervals with little difference between each other. High and low WR indicates low and high temporal sensitivity, respectively^47^.

#### Prismatic adaptation

To ensure that pre-PA/post-PA differences in time bisection task were due to the PA procedure we assessed the presence of both error reduction and aftereffect.

To verify that participants showed *error reduction*, we conducted an ANOVA, for each prismatic deviation, on the mean displacement (expressed as degrees of visual angle) of participants’ visible pointing, with Age (early-children, children and late-children) as a between-group variable and Condition (pre-exposure condition, first three trials of the exposure condition, last three trials of the exposure condition) as a within-subjects variable (more details on this procedure^3^).

To verify the presence of an *aftereffect*, we compared participants’ displacement during invisible pointing in the pre-exposure and post-exposure conditions. An ANOVA, for each aftereffect direction, was conducted on the mean displacement of invisible pointing responses with Age (early-children, children and late-children) as a between-group variable and Condition (pre-exposure vs post-exposure) as a within-subjects variable.

Post-hoc comparisons, if necessary, were conducted using the Duncan test. Effect size is reported as partial eta square.
